# Differences in healthy life expectancy between older migrants and non-migrants in three European countries over time

**DOI:** 10.1007/s00038-017-0949-6

**Published:** 2017-02-26

**Authors:** Matias Reus-Pons, Eva U. B. Kibele, Fanny Janssen

**Affiliations:** 1 Population Research Centre, Faculty of Spatial Sciences, University of Groningen, Groningen, The Netherlands; 2 Interface Demography, Department of Sociology, Vrije Universiteit Brussel, Brussels, Belgium; 3 Netherlands Interdisciplinary Demographic Institute (NIDI), The Hague, The Netherlands

**Keywords:** Health, Mortality, Migration, Ageing, Belgium, The Netherlands, England and Wales

## Abstract

**Objectives:**

We analysed differences in healthy life expectancy at age 50 (HLE_50_) between migrants and non-migrants in Belgium , the Netherlands, and England and Wales, and their trends over time between 2001 and 2011 in the latter two countries.

**Methods:**

Population, mortality and health data were derived from registers, census or surveys. HLE_50_ and the share of remaining healthy life years were calculated for non-migrants, western and non-western migrants by sex. We applied decomposition techniques to answer whether differences in HLE_50_ between origin groups and changes in HLE_50_ over time were attributable to either differences in mortality or health.

**Results:**

In all three countries, older (non-western) migrants could expect to live less years in good health than older non-migrants. Differences in HLE_50_ between migrants and non-migrants diminished over time in the Netherlands, but they increased in England and Wales. General health, rather than mortality, mainly explained (trends in) inequalities in healthy life expectancy between migrants and non-migrants.

**Conclusions:**

Interventions aimed at reducing the health and mortality inequalities between older migrants and non-migrants should focus on prevention, and target especially non-western migrants.

## Introduction

While the issues of migration, ageing, and health are on the political agenda in all European countries, little attention has been paid to the health of older migrants in Europe (Rechel et al. 2011). Studying health and mortality among older migrants in Europe is important because the share of older migrants in European populations is rising steadily (Lanzieri 2011). Addressing potential health disparities between older migrants and non-migrants is consistent with the principle of equity embedded in most European health care systems and policies (Nørredam and Krasnik 2011). Knowledge about the health of older migrants will prove crucial in assessing future health care demand in culturally diverse and ageing populations (International Organization for Migration 2009), and to inform policies and interventions.

Earlier studies on migrant health and mortality produced different results. Despite their relatively low socio-economic status, certain migrant groups have been shown to live longer than non-migrants; this phenomenon is described as the migrant mortality paradox (e.g. Razum et al. 1998; Abraído-Lanza et al. 1999). Even when an overall migrant mortality advantage is not observed, migrants may still have a mortality advantage compared with non-migrants in a similar socio-economic position (Riosmena et al. 2013). However, living longer does not necessarily imply living in good health (Uitenbroek and Verhoeff 2002). Indeed, migrants tend to have worse self-rated health than non-migrants (Nielsen and Krasnik 2010).

The few existing studies that focused on this issue found that health and mortality differences between migrants and non-migrants persist with age. At older ages, migrants tend to have lower mortality than non-migrants (Markides and Eschbach 2005; Carnein et al. 2014; Lariscy et al. 2015; Reus-Pons et al. 2016), but also worse self-rated health, worse functioning, and higher rates of disability and depression (Solé-Auró and Crimmins 2008; Lanari and Bussini 2012; Carnein et al. 2014). While previous studies showed that migrants tend to experience a steeper decline in health with age and length of stay (Ronellenfitsch and Razum 2004; Lanari and Bussini 2012), this was not the case for mortality (Markides and Eschbach 2005; Reus-Pons et al. 2016).

To address the questions surrounding the health and mortality differences between migrants and non-migrants, the combined study of health and mortality is essential. Healthy life expectancy (HLE) is a powerful tool for tackling these issues, and can be used to make cross-country comparisons. However, earlier cross-country comparisons of HLE did not break down the population by migrant origin (Jagger et al. 2008, 2011; Wohland et al. 2014; Fouweather et al. 2015); and to our knowledge, only one existing study has applied HLE in studying health and mortality differences between older migrants and non-migrants in a single country (Carnein et al. 2014).

Moreover, as health inequalities between countries (Fouweather et al. 2015) and between socio-economic groups (Hu et al. 2016) are growing, evaluating the trends in the HLE gaps between older migrants and non-migrants could provide us with answers to the question of whether health inequalities between migrants and non-migrants (subsequently referred to as migrant health inequalities) are also increasing or, in contrast, decreasing. Up to now, the only studies on this issue that incorporated a time dimension did not break down the population by migrant origin (Wohland et al. 2014; Fouweather et al. 2015; Hu et al. 2016).

Our aim is to compare the differences in HLE between older migrants and non-migrants in three European countries: Belgium, the Netherlands, and England and Wales; and to assess their trends over time in the latter two countries.

We selected these three countries because they have similar life expectancies at birth, similar migration histories, and reliable data. The vast majority of the older migrants living in Europe today are first-generation migrants who arrived before the early 1970s as guest workers, from neighbouring countries, or from former colonies (Lanzieri 2011). However, the largest country of origin groups differ in each of these three countries due to different colonial ties, and to the fact that labour migrants originated from different areas (Mediterranean countries in Belgium and the Netherlands, and New Commonwealth countries in England and Wales).

## Methods

### Data

In this study, we focus on first-generation migrants and non-migrants aged 50 years and over in Belgium (2001), the Netherlands (2001 and 2011), and England and Wales (2001 and 2011). Migrants were defined as those born in a country other than their current country of residence. According to their country of origin, migrants were then subdivided into western (origin in a European country, USA, Canada, Australia, New Zealand, or Japan) and non-western (CBS 2016a). In England and Wales, individuals born in other parts of the UK were also classified as western migrants.

To calculate healthy life expectancy at age 50 (HLE_50_)—i.e. the expected number of remaining years spent in good health—we relied on yearly population, mortality, and health data by sex, migrant origin, and five-year age groups (50–54, …, 85+), which were derived from registers, censuses, and surveys we obtained from Statistics Belgium, Statistics Netherlands, and the Office for National Statistics (Table 1).

**Table 1:** Data sources by country and year

Country	Year	Population		Deaths		Self-rated health	
		Source	Year	Source	Year	Source	Year
Belgium	2001	Census	2001	Register	2002	Census^a^	2001
The Netherlands	2001	Register	2001	Register	2001	Survey data: Permanent Survey on the Living Situation (POLS) and Health Survey^b^	2001^c^
	2011		2011		2011		2011^c^
England and Wales	2001	Census	2001	Death certificates	2001	Census	2001
	2011		2011		2011		2011

^a^Data from the Belgian Health Interview Survey not used due to the large amount of missing data

^b^The Health Survey substituted the part on health of the POLS after 2009, but no major changes were made to the question and answer choices regarding self−rated health

^c^Data from the different surveys in the Netherlands were aggregated around the years 2001 (POLS 1999−2003) and 2011 (POLS 2009, Health Survey 2010−2013) to increase the sample size

We reclassified self-rated health from its original five categories (very good, good, fair, bad, very bad) to a binary variable, distinguishing between good (good to very good) and poor health (very bad to fair). In the 2001 census for England and Wales only, self-rated health was originally classified in three categories instead (good, fairly good, not good). To allow for comparability, we applied adjustment factors developed by ONS (Smith and White 2009).

The Dutch survey data were weighted by Statistics Netherlands based on age, sex, and other demographic characteristics, including migrant background (CBS 2016b, c) to represent the national population. In Belgium, data on self-rated health was missing for around 5% of the non-migrant population and around 10% of the migrant population; we therefore weighted the Belgian self-rated health data using simple ratio weights (Fawcett et al. 2002) based on sex, age, migrant background, education, and urbanity of the area of residence.

In 2001, the proportion of migrants who were aged 50 and over was 11.4% in England and Wales, 11.1% in Belgium and 7.6% in the Netherlands (Table 2). The majority of older migrants in all three countries were of western origin. However, in 2011 the majority of male migrants in the Netherlands and in England and Wales were of non-western origin. Individuals born in other parts of the UK constituted 23.9% (2001) and 19.5% (2011) of the migrant population in England and Wales.

**Table 2:** Population aged 50 and over (*N*_50+_), and sample size in the health survey (*n*_50+_) by sex, migrant origin, and country in Belgium (2001), the Netherlands (2001–2011), and England and Wales (2001–2011)

	Belgium	The Netherlands				England and Wales	
	2001	2001		2011		2001	2011
	*N*_50+_	*N*_50+_	*n*_50+_	*N*_50+_	*n*_50+_	*N*_50+_	*N*_50+_
Males							
Total	1,587,355	2,306,401	24,637	2,842,126	12,369	7,991,367	9,114,457
Non-migrants	1,407,572	2,129,003	23,132	2,584,237	11,581	7,075,198	7,904,468
Migrants	179,783	177,398	1505	257,889	788	916,169	1,209,989
Western migrants	137,501	98,962	1004	114,573	426	542,579	598,162
Non-western migrants	42,282	78,436	501	143,316	362	373,590	611,827
Females							
Total	1,915,005	2,667,522	26,317	3,143,038	13,340	9,419,478	10,271,387
Non-migrants	1,705,610	2,467,807	24,671	2,854,149	12,476	8,344,831	8,853,063
Migrants	209,395	199,715	1646	288,889	864	1,074,647	1,418,324
Western migrants	173,509	128,682	1222	144,625	511	674,695	742,971
Non-western migrants	35,886	71,033	424	144,264	353	399,952	675,353

Data sources: Statistics Belgium, Statistics Netherlands, and Office for National Statistics© Crown Copyright 2015

### Methods

HLE_50_ was calculated using the Sullivan method (1971). To test whether there were differences in HLE_50_ between older migrants and non-migrants, we calculated 95% confidence intervals (Jagger et al. 2006). Additionally, to provide a full picture, we estimated the proportion of the expected remaining years of life spent in good health (HLE_50_/LE_50_), where LE_50_ stands for life expectancy at age 50, calculated using standard life table techniques (Preston et al. 2000).

Trends in HLE_50_ by migrant background over time were assessed by comparing both changes in HLE_50_ and in HLE_50_/LE_50_ between 2001 and 2011. Decomposition techniques were applied to identify to what extent the differences in HLE_50_ between groups and the changes in HLE_50_ over time were attributable to differences in mortality, or to differences in self-rated health (Nusselder and Looman 2004).

## Results

### Inequalities in HLE_50_ between migrants and non-migrants

Regardless of the fact that migrants’ LE_50_ was higher than that of non-migrants in Belgium (2001), and in the Netherlands and England and Wales (2011), HLE_50_ was significantly lower for migrants, especially those of non-western origin, than for non-migrants in all three countries and in both 2001 and 2011 (Table 3). The largest migrant inequality gap in HLE_50_ was found in the Netherlands. The estimated proportion of the expected remaining years of life spent in good health (HLE_50_/LE_50_) followed a similar pattern. In England and Wales only, western migrants could expect to live a larger share of their remaining life in good health than non-migrants in both 2001 and 2011.

**Table 3:** Life expectancy (LE_50_) and healthy life expectancy (HLE_50_) at age 50, and share of years spent in good health after age 50 (HLE_50_/LE_50_) by sex and migrant origin in Belgium (2001), the Netherlands (2001–2011), and England and Wales (2001–2011)

	Belgium (2001)			The Netherlands (2001)			England and Wales (2001)		
	LE_50_ (years)	HLE_50_ (95% CI) (years)	HLE_50_/LE_50_	LE_50_ (years)	HLE_50_ (95% CI) (years)	HLE_50_/LE_50_	LE_50_ (years)	HLE_50_ (95% CI) (years)	HLE_50_/LE_50_
Males									
Total	27.87	14.48 (14.44, 14.52)	0.519	28.05	18.62 (18.40, 18.84)	0.664	28.54	18.47 (18.46, 18.48)	0.647
Non-migrants	27.79	14.71 (14.66, 14.75)	0.529	28.15	18.92 (18.69, 19.14)	0.672	28.66	18.59 (18.58, 18.60)	0.649
Migrants	28.26	12.52 (12.41, 12.62)	0.443	26.77	14.88 (14.03, 15.72)	0.556	27.52	17.53 (17.50, 17.55)	0.637
Western migrants	28.14	12.61 (12.49, 12.73)	0.448	26.60	17.12 (16.16, 18.07)	0.644	27.06	17.61 (17.58, 17.65)	0.651
Non-western migrants	29.52	12.32 (12.03, 12.60)	0.417	27.78	10.57 (8.72, 12.42)	0.381	28.26	17.43 (17.38, 17.48)	0.617
Females									
Total	32.84	15.29 (15.25, 15.32)	0.465	32.47	19.43 (19.19, 19.68)	0.599	32.32	19.82 (19.81, 19.83)	0.613
Non-migrants	32.82	15.61 (15.57, 15.64)	0.475	32.58	19.76 (19.51, 20.02)	0.607	32.39	19.94 (19.93, 19.95)	0.616
Migrants	33.16	12.76 (12.66, 12.85)	0.385	31.01	15.23 (14.31, 16.15)	0.491	31.82	19.02 (18.99, 19.05)	0.598
Western migrants	33.31	13.16 (13.05, 13.26)	0.395	30.91	17.12 (16.06, 18.18)	0.554	31.68	19.78 (19.74, 19.82)	0.624
Non-western migrants	33.55	11.51 (11.19, 11.82)	0.343	32.13	11.60 (9.67, 13.53)	0.361	32.13	17.83 (17.77, 17.88)	0.555

	Belgium (2011)			The Netherlands (2011)			England and Wales (2011)		
	LE_50_ (years)	HLE_50_ (95% CI) (years)	HLE_50_/LE_50_	LE_50_ (years)	HLE_50_ (95% CI) (years)	HLE_50_/LE_50_	LE_50_ (years)	HLE_50_ (95% CI) (years)	HLE_50_/LE_50_
Males									
Total	–	–	–	30.87	20.83 (20.55, 21.10)	0.675	31.29	18.71 (18.70, 18.72)	0.598
Non-migrants	–	–	–	30.95	21.09 (20.80, 21.37)	0.681	31.32	18.82 (18.81, 18.83)	0.601
Migrants	–	–	–	29.88	17.18 (15.99, 18.37)	0.575	31.01	17.98 (17.95, 18.01)	0.580
Western migrants	–	–	–	29.38	18.79 (17.36, 20.22)	0.640	30.04	18.26 (18.22, 18.29)	0.608
Non-western migrants	–	–	–	31.13	15.20 (12.68, 17.72)	0.488	32.31	17.72 (17.68, 17.77)	0.548
Females									
Total	–	–	–	34.31	20.68 (20.37, 20.99)	0.603	34.50	19.67 (19.66, 19.68)	0.570
Non-migrants	–	–	–	34.36	20.95 (20.63, 21.27)	0.610	34.47	19.89 (19.87, 19.90)	0.577
Migrants	–	–	–	33.49	16.77 (15.47, 18.06)	0.501	34.84	18.41 (18.39, 18.44)	0.528
Western migrants	–	–	–	33.05	18.43 (16.94, 19.92)	0.558	34.33	20.02 (19.98, 20.05)	0.583
Non-western migrants	–	–	–	34.73	14.49 (11.44, 17.54)	0.417	35.71	16.53 (16.48, 16.58)	0.463

Data sources: Statistics Belgium, Statistics Netherlands, and Office for National Statistics© Crown Copyright 2015

Migrant inequalities in HLE_50_ were mainly attributable to differences in self-rated health (Table 4). Mortality often contributed in the opposite direction; for example, for Belgian males in 2001, the negative contribution of mortality was due to the lower overall mortality among migrants. In contrast to the general trend, migrant inequalities in HLE_50_ in England and Wales were mainly explained by differences in mortality, since western migrants, albeit experiencing higher mortality, could expect to live a larger share of their remaining life in good health than non-migrants.

**Table 4:** Decomposed differences in healthy life expectancy at age 50 (HLE_50_) between migrant origin groups by sex in Belgium (2001), the Netherlands (2001–2011), and England and Wales (2001–2011)

	2001			2011		
	Difference in HLE_50_ (years)	Difference due to		Difference in HLE_50_ (years)	Difference due to	
		Mortality (%)	Self-rated health (%)		Mortality (%)	Self-rated health (%)
*Difference between non-migrants and migrants*						
Males						
Belgium	2.19*	−9.6	109.6	–	–	–
The Netherlands	4.04*	19.1	80.9	3.90*	15.9	84.1
England and Wales	1.06*	61.2	38.8	0.84*	18.7	81.3
Females						
Belgium	2.85*	−3.8	103.8	–	–	–
The Netherlands	4.53*	17.7	82.3	4.19*	9.3	90.7
England and Wales	0.93*	29.3	70.7	1.47*	−8.7	108.7
*Difference between non-migrants and western migrants*						
Males						
Belgium	2.10*	−8.1	108.1	–	–	–
The Netherlands	1.80*	49.6	50.4	2.29*	41.1	58.9
England and Wales	0.97*	94.1	5.9	0.56*	105.3	−5.3
Females						
Belgium	2.45*	−6.7	106.7	–	–	–
The Netherlands	2.64*	32.8	67.2	2.52*	24.6	75.4
England and Wales	0.16*	214.7	−114.7	−0.13*	−63.2	163.2
*Difference between non-migrants and non-western migrants*						
Males						
Belgium	2.39*	−21.5	121.5	–	–	–
The Netherlands	8.34*	1.9	98.1	5.88*	−1.5	101.5
England and Wales	1.15*	20.9	79.1	1.10*	−32.5	132.5
Females						
Belgium	4.10*	−3.1	103.1	–	–	–
The Netherlands	8.16*	2.6	97.4	6.46*	−3.3	103.3
England and Wales	2.12*	5.4	94.6	3.36*	−11.9	111.9
*Difference between western migrants and non-western migrants*						
Males						
Belgium	0.29	−119.4	219.4	–	–	–
The Netherlands	6.55*	−8.1	108.1	3.59	−27.6	127.6
England and Wales	0.18*	−356.3	456.3	0.53*	−172.6	272.6
Females						
Belgium	1.65*	0.5	99.5	–	–	–
The Netherlands	5.52*	−11.3	111.3	3.94	−24.3	124.3
England and Wales	1.96*	−11.6	111.6	3.49*	−13.8	113.8

Data sources: Statistics Belgium, Statistics Netherlands, and Office for National Statistics© Crown Copyright 2015

*Statistically significant (*p* < 0.05)

### Trends in HLE_50_ between 2001 and 2011

Between 2001 and 2011, the gap in HLE_50_ between (non-western) migrants and non-migrants diminished in the Netherlands and among males in England and Wales, but widened among females in England and Wales (Table 3). However, if we look at the change in HLE_50_/LE_50_, we see that migrant health inequalities increased for both sexes in England and Wales. Although non-western migrants continued to be the group with the lowest HLE_50_ and HLE_50_/LE_50_ in the Netherlands, the gap with respect to non-migrants and western migrants decreased slightly.

In general, we find that increases in HLE_50_ were mainly attributable to decreases in mortality, and were driven by improvements in self-rated health only among non-western migrants in the Netherlands (Table 5). Improvements in HLE did not keep pace with improvements in LE for most groups. The decreases in HLE_50_ among females in England and Wales were driven by declines in the prevalence of good self-rated health.

**Table 5:** Decomposed change in healthy life expectancy at age 50 (HLE_50_) between 2001 and 2011 by sex and migrant origin in the Netherlands and in England and Wales (2001–2011)

	The Netherlands			England and Wales		
	Difference in HLE_50_ (years)	Difference due to		Difference in HLE_50_ (years)	Difference due to	
		Mortality (%)	Self-rated health (%)		Mortality (%)	Self-rated health (%)
Males						
Total	2.21*	75.4	24.6	0.25*	533.0	−433.0
Non-migrants	2.17*	76.7	23.3	0.23*	547.2	−447.2
Migrants	2.30*	72.6	27.4	0.45*	371.5	−271.5
Western migrants	1.67	95.0	5.0	0.64*	232.4	−132.4
Non-western migrants	4.63*	30.1	69.9	0.29*	636.6	−536.6
Females						
Total	1.25*	71.3	28.7	−0.15*	−609.9	709.9
Non-migrants	1.19*	71.6	28.4	−0.06*	−1502.9	1602.9
Migrants	1.54	80.5	19.5	−0.60*	−205.9	305.9
Western migrants	1.31	83.3	16.7	0.24*	473.3	−373.3
Non-western migrants	2.89	41.8	58.2	−1.30*	−103.7	203.7

Data sources: Statistics Netherlands, and Office for National Statistics© Crown Copyright 2015

*Statistically significant (*p* < 0.05)

## Discussion

### Summary of the results

In all three countries studied, migrants aged 50 years and older could expect to live fewer years in good self-rated health than non-migrants. Non-western migrants had the lowest HLE_50_, especially in the Netherlands. The differences in HLE_50_ between (non-western) migrants and non-migrants were mainly determined by differences in self-rated health. Between 2001 and 2011, migrant inequalities in both HLE_50_ and HLE_50_/LE_50_ were reduced in the Netherlands, mainly driven by improvements in self-rated health among non-western migrants. While migrant inequalities in HLE_50_ diminished among males in England and Wales, migrant inequalities in HLE_50_/LE_50_ increased for both sexes.

### Evaluation of the data and methods

The results of our analysis are based on highly reliable population and health data. Nevertheless, several limitations of the study should be noted. Although self-rated health has been reported to be reliable for the total population, concerns have been raised about its use when comparing different ethnic groups (e.g. Chandola and Jenkinson 2000). Seo et al. (2014), however, found that variations in the response patterns do not differ according to origin, but to the responding language instead. In our study, census and surveys were provided in the national languages only, which helps reducing the potential variability in the response pattern between migrants and non-migrants. Furthermore, studies of older migrants that relied on more objective health indicators, such as depression, functioning, or disability (Solé-Auró and Crimmins 2008; Lanari and Bussini 2012; Carnein et al. 2014), found similar results, i.e. migrants are less healthy than non-migrants.

Our data might also suffer from comparability issues between countries and over time. Even when the same question format is used, self-rated health outcomes reported by the older population in surveys may vary due to differences in survey response, sample size, and survey mode (Croezen et al. 2016). For instance, the exclusion of people living in institutions from the sample frame in the Netherlands might have led to an overestimation of HLE_50_, as a high share of the population—and especially non-migrants and western migrants—live in institutions after age 80. In England and Wales, the self-rated health data in the 2001 census were originally classified in three response categories instead of five. Although we applied adjustment factors to ensure comparability across countries and over time, the adjustment factors are less reliable among the oldest old (Smith and White 2009). To assess the influence of these data limitations on our HLE_50_ estimations, we performed a sensitivity analysis excluding the population aged 80 and over. We therefore calculated the temporary healthy life expectancy between ages 50 and 79 (THLE_50−79_) by applying the Sullivan method (1971) to the temporary life expectancy (Arriaga 1984) between ages 50 and 79 (results not shown). The most remarkable difference found in both analyses was that the THLE_50−79_ for females in England and Wales increased between 2001 and 2011, while the HLE_50_ decreased. The THLE_50−79_ gap between non-migrants and non-western migrants in the Netherlands was also smaller than the HLE_50_ gap; thus supporting the assumption that the large migrant health inequalities in the Netherlands were, at least partially, attributable to the exclusion of the institutionalized population from the sample frame. Nevertheless, similar patterns were found in HLE_50_ and THLE_50−79_ when comparing migrants and non-migrants across countries, and when comparing trends over time. In light of the outcomes of these additional THLE_50−79_ analyses, we may not be able to identify with certainty the countries in which older migrants have a longer or a shorter HLE_50_. However, we can conclude that older migrants, especially those of non-western origin, can expect to live fewer years in good self-rated health than older non-migrants, in all three countries studied. These findings are consistent across the three countries, and with the results of previous studies in Europe (Solé-Auró and Crimmins 2008; Lanari and Bussini 2012). In a similar vein, while we may be unable to state with certainty that HLE_50_ among females in England and Wales decreased over time, we can assert that the migrant health gaps in HLE_50_ and HLE_50_/LE_50_ in England and Wales increased.

Finally, we classified residents of England and Wales who were born in other parts of the UK as western migrants. Since the migration trajectories of these internal migrants are likely to differ considerably from those of international migrants, we performed a sensitivity analysis in which we excluded Scottish and Northern Irish individuals from the dataset. This did not substantially alter the results, and the conclusions drawn from the comparison of HLE_50_ and HLE_50_/LE_50_ between groups and over time remained the same (results not shown).

### Interpretation of the results

Using HLE as an indicator that combines mortality and health, our results consistently show that the HLE of older migrants, especially those of non-western origin, was lower than that of non-migrants. In most cases, these differences were mainly attributable to differences in self-rated health. Thus, our results are consistent with those of previous studies that merely used health as an outcome measure. These studies showed that compared to their non-migrant counterparts, older migrants in Europe have worse self-rated health, and more chronic conditions, limitations, and depression (Solé-Auró and Crimmins 2008; Lanari and Bussini 2012; Carnein et al. 2014). Poor health among migrants has often been explained by a range of individual and contextual factors, including economic difficulties, poor housing and working conditions, limited access to health care, cultural and language barriers, and social exclusion (Gushulak et al. 2010). The health-related lifestyles migrants adopt over their life course can affect their health at older ages; in addition, older migrants may be more prone than non-migrants to contracting diseases related to early life deprivation in their country of origin (Razum and Twardella 2002). The results also indicate, however, that the contribution of mortality to differences in HLE_50_ between migrants and non-migrants was often small, and in certain cases, even contributed in the opposite direction. These findings are in line with the general migrant mortality paradox (Razum et al. 1998; Abraído-Lanza et al. 1999), or at least with weaker versions of it (Riosmena et al. 2013). The decomposition results illustrate how health and mortality do not necessarily follow a similar pattern, and hence the added value of using a combined measure (HLE) to study health and mortality disparities between migrants and non-migrants.

In England and Wales, the migrant HLE_50_ inequalities decreased among males, but increased among females. However, the migrant HLE_50_/LE_50_ inequality gap in England and Wales increased for both sexes. The discrepancy among males can be attributed to the failure of improvements in HLE to keep pace with improvements in LE (morbidity expansion), especially among non-western migrants. Previous studies have also found that contemporary improvements in HLE in Europe tend to be slower than improvements in LE (Harper 2015). The increase in migrant HLE inequalities observed in England and Wales follows more general patterns, such as the increase in differences in HLE between local areas in Great Britain (Wohland et al. 2014) or between European countries (Fouweather et al. 2015). Economic hardship due to the economic crisis may explain why self-rated health did not improve over time (Clair et al. 2016), especially among (non-western) migrants, who are especially vulnerable to economic downturns given their fragile socio-economic position (International Organization for Migration 2010).

Our results also show, however, that migrant inequalities in both HLE_50_ and in HLE_50_/LE_50_ in the Netherlands declined over time. In fact, only among non-western migrants in the Netherlands, improvements in HLE_50_ over time were mainly driven by improvements in self-rated health, rather than by decreases in mortality. Although non-western migrants were the only group in the Netherlands for whom improvements in HLE were markedly faster than improvements in LE, there was also no morbidity expansion among western migrants or non-migrants either. A potential explanation for this finding is that unlike in most European countries, public spending on health in the Netherlands after the 2008 crisis was increased, and measures aimed at reducing pressure on highly congested medical services were implemented (Mladovsky et al. 2012).

### Overall conclusion

Our analysis of health and mortality differences between older migrants and non-migrants across three countries over a 10-year period has generated some important new findings. Self-rated health, rather than mortality, seems to be the key explanatory factor beyond migrant inequalities in HLE, and their reduction over time. Interventions to reduce the health and mortality inequalities between older migrants and non-migrants should focus mainly on prevention rather than (palliative) treatment, and target the most disadvantaged groups, including non-western migrants.
